# Evaluating the severity of microvascular invasion in hepatocellular carcinoma, by probing the combination of enhancement modes and growth patterns through magnetic resonance imaging

**DOI:** 10.2478/raon-2025-0021

**Published:** 2025-04-11

**Authors:** Yanzhuo Li, Sijie Li, Yan Lei, Lianlian Liu, Bin Song

**Affiliations:** 1 Department of Radiology, Minhang Hospital, Fudan University, Shanghai, People’s Republic of China; 2 Department of Radiology, Changhai Hospital, Naval Medical University, Shanghai, People’s Republic of China

**Keywords:** hepatocellular carcinoma, microvascular invasion, stratified prediction, magnetic resonance imaging, nomograms

## Abstract

**Background:**

Microvascular invasion (MVI), particularly its severity, correlates with prognosis in hepatocellular carcinoma (HCC), however, it remains uncertain which imaging traits are associated with MVI grades. Predicting MVI status precisely pre-surgery assists clinicians in making optimal treatment decisions.

**Patients and methods:**

213 HCC patients with surgically confirmed were assigned into three groups based on the severity of MVI (M0, M1, and M2). Clinical and imaging features were compared between each group. Univariate and multivariate analyses were used to identify the significant variables associated with MVI severity. Subsequently, nomograms were constructed to estimate MVI and its M2 grade by crucial factors. Nomograms were assessed for accuracy, clinical value, and efficacy using the area under the curve (AUC), calibration curve, and decision curve analysis (DCA).

**Results:**

Four factors associated with MVI (P < 0.05) were related, including non-solitary growth types, no/mini enhanced mode, peritumoral enhancement on arterial phase, and peritumoral hypointensity on hepatobiliary phase. Only the ratio of the maximum and minimum tumor diameter (Max/Min-R), confluent multinodule growth type, and non-washin/washout enhanced modes of those MVI-positive patients showed a strong correlation with M2 grade. The areas under the receiver operating characteristic (ROC) curves were 0.885 (95% confidence intervals [CI]: 0.833–0.937) in identifying MVI and 0.805 (95% CI: 0.703–0.908) in predicting its M2 grade, respectively. The nomograms demonstrated a high goodness-of-fit and clinical benefits in DCA and calibration curve.

**Conclusions:**

Enhancement modes and tumor growth patterns of preoperative MRI were independent risk factors of MVI severity, which were valuable for facilitating individualized decision-making.

## Introduction

Hepatocellular carcinoma (HCC) is one of the top lethal malignancies worldwide with the largest HCC burden in our country, even if new cases are diagnosed and cancer-related deaths are yearly reduced.^1^ Though steady advances in screening and clinical strategies improve early detection and systemic therapies, the survivals remain undesirable due to the high incidence of recurrence and metastasis.^1,2^ Microvascular invasion (MVI) should be one of the primary responsibilities for long-term survival and an explicit risk factor for relapse.^3^ According to the findings^4,5^, not only the presence of MVI, in particular, severity grades closely correlate with long-term recurrence and cause varying prognoses. In general, frequent tests and intense interventions are necessary for patients’ duration, however, they are not indicated for all grades of MVI. Research showed that compared with low-risk MVI, high-risk patients sustain a higher rate of recurrence of 86.1% and a lower rate of overall survival of 67% in 3-year postoperative.^4,6^ Therefore, patients who have severe MVI stage require more aggressive treatment procedures and sophisticated follow-up protocols, that theoretically and effectively reduce the rate of recurrence after treatment and favor prognosis.^7,8^ To assess the severity of MVI, accurate detection at several points is needed in postoperative pathological specimens and is hardly applied in clinical practice before surgery, due to unreliably diagnosed with limited details and some repulsive drawbacks.^8,9^ Besides, no non-invasive option exists currently that can surpass the “gold standard” of histological specimens in MVI detection or categorization.^10^

Thanks to the inclusion of sequences and parameters, magnetic resonance imaging (MRI) has superiorities in detecting and evaluating the status of MVI, which compensates for the absence of preoperative assessment by biopsy, in which hepatobiliary contrast agents play an essential role as well^11-13^, it increased the sensitivity of diagnosing small HCCs that depicted non-arterial enhancing mode.^14^ Gadolinium ethoxybenzyl diethylenetriamine pentaacetic acid (GD-EOB-DTPA) enhanced MRI has been reported as useful for assessing the microstructure and particular signs of the lesion.^15^ Hitherto, certain imaging features have been defined as characteristic of the MVI-positive, and a portion of the literature has described and explored MVI stratification, however, no consensus has been achieved, and no distinctive imaging findings can be graded to determine the severity of MVI.^12,16^ Thus preoperative assessment of MVI status is required in selecting the appropriate surgical method and establishing suitable therapy in different durations.^17^ Although radiomics offers several benefits, the operations are relatively complex, and the predictive results are indirect, lacking validation, and unsuitable for routine clinics which unequipped with specialized machines and software.

That is to say, a reusable, application-friendly noninvasive test and predictive model need to be developed to identify the severity of MVI and substantial advantages for patients in the selection of preoperative surgical procedures.^18^ Nevertheless, no research has considered a combination of tumor growth patterns and enhancement modes to develop a nomogram model that can predict MVI grade. Hence, we extract clinicoradiological factors and imaging descriptions that can be clinically valuable for detecting MVI existence and its grade and establish a fusion model for preoperative evaluation.

## Patients and methods

### Study participants

Since all the tests were routine protocols before surgery, neither unique contrast agents nor unusual sequences were used. The retrospective study was approved by the hospital ethics committees and waived patient informed consent. Between January 2019 and January 2022, by searching the pathological database, patients included were as follows: (a) Pre-operative GD-EOB-DTPA enhanced MRI examination, (b) Pathologically proven HCC. Patients were excluded according to the following criteria: (a) Recurrence HCCs or with other previous malignancies history, (b) Unsatisfied MRI images with severe artifacts in enhancement imaging, (c) Antitumor therapy before MRI examination, (d) MRI acquired more than 1 month before hepatectomy, (e) Radiographically visible extrahepatic lesions, macrovascular invasion, or lymphatic invasion, (f) Tumor size smaller than 1 cm, (g) Nonthorough clinical, laboratory, imaging, and pathological data, especially incomplete assessment of MVI description. Overall, the final cohort consisted of 213 consecutive patients (177 men and 36 women; mean age, 57.4 ± 0.8 years) (Supplementary Figure 1).

### Liver MRI protocol

MRI was performed with acquired with various 1.5-T (Signa Explorer, GE Healthcare) or 3.0-T (Discovery MR750, GE Healthcare) scanners. Routine liver MRI protocols were performed with the following sequences: T2-weighted imaging, T1-weighted imaging, and diffusion-weighted imaging. Dynamic imaging was performed with a T1-weighted fat-suppressed sequence. GD-EOB-DTPA -enhanced imaging (Primovist; Bayer Berlin, Germany) for a dose of 0.1 mmoL/kg was injected into patients’ median cubital veins at a flow rate of 2.0 mL/s with a high-pressure syringe, followed by a 20 ml saline flush. Dynamic contrast-enhanced imaging of the arterial phase (AP), portal venous phases (PVP), and delayed phases (DP), were acquired at 22–25s, 50–60s, and 90–120s after injecting gadoxetic acid, respectively. Hepatobiliary phase (HBP) acquisition was performed about 10-20 mins after injection, depending on the patient’s age, weight, etiology, and Child-Pugh classification. And axial T1-weighted three-dimensional (3D) GRE was obtained with fat suppression (Liver Acquisition with Volume Acceleration, LAVA). The detailed parameters are presented in Supplementary Table 1.

### Qualitative MRI features evaluation

Two radiologists (S.J.L. and Y.L., with 14 and 20 years of experience in abdominal imaging, respectively) retrospectively retrieved the MR images from the Picture Archiving and Communication Systems (PACS) and reviewed them separately. After being aware of the diagnosis of HCC, they assessed randomly with all images de-labeled (sex, age, etc.) and the clinical, laboratory, and pathological data blinded. For patients with multiple lesions, the reviewers assessed the main tumor for feature-based analyses. The reviewers also independently evaluated tumor hallmarks as follows:

(A) Basic features of HCC: (1) tumor size, including the ratio of the maximum and minimum tumor diameter (Max/Min-R), (2) tumor shape, (round or oval shape, multi-nodules or irregular budding) (Figure 2 D, L)^19^; (3) tumor margin, (clear or unclear) (Figure 2 D, H); (4) Tumor growth morphology, (Solitary nodule, Extranodular nodule, Confluent multinodule, Massive, Immersed).^20^
(B) Known MRI features associated with MVI: (5) arterial rim-enhancement, means irregular rim-like peripheral hyperenhancement with a central hypoenhancing area in AP^21^; (6) arterial peritumoral enhancement on AP, defined as a detectable portion of crescent or polygonal shaped enhancement outside the tumor boundary on AP, and being iso-intense to background liver parenchyma on DP^21^; (7) peritumoral hypointensity on HBP, manifested as wedge-shaped or flame-like area outside the tumor with a lower intensity than liver parenchyma but a higher intensity than the tumor on HBP^21^; (8) beak sign, shows as tumors with slightly sharp margins at an acute angle to the liver parenchyma.^22^
(C) Diagnostic features of HCC: (9) radiologic capsule, shows a rim of smooth hyperenhanced structure around the tumor during PVP or DP (Figure 2 L)^18^; (10) Dynamic enhancement modes (Arterial enhancement with washout on PVP or DP[washin/washout]; Arterial and persistent enhancement; Gradual enhancement; No or minimal enhancement).^20^

**Figure 1.**
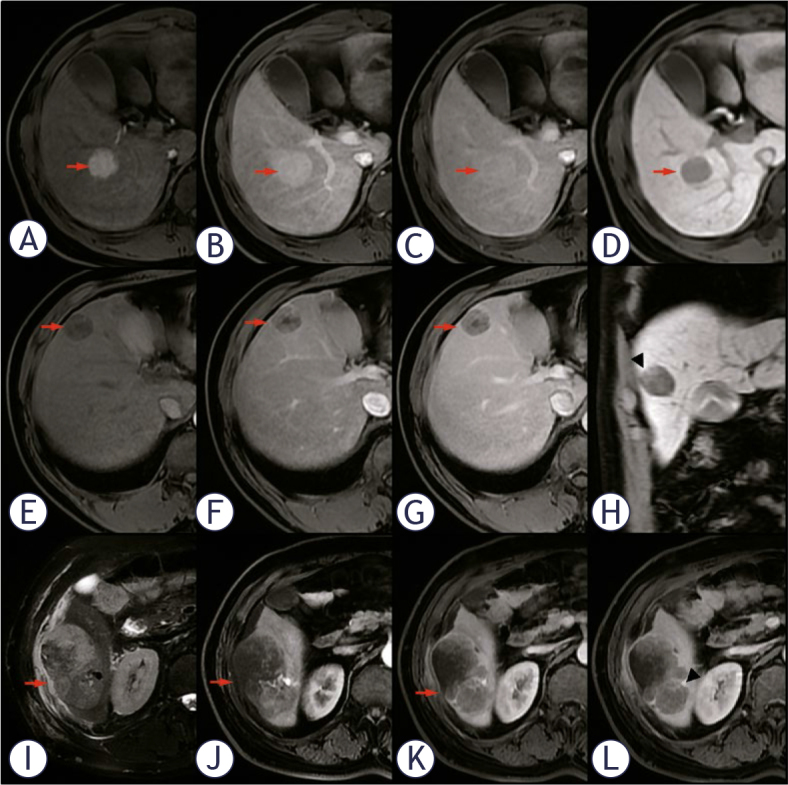
A 34-year-old male with a clearly bounded solitary HCC and M0 status, **(A-D)** exhibits persistent enhancement in AP, PVP, DP, and a smooth tumor margin (red arrow). A 52-year-old male with HCC and M1 grade, which illustrates a typical no/mini enhancement (red arrow) **(E-G)**. Vague margin is visible at the superior edge of the tumor (black arrowhead) **(H)**. A 65-year-old male who had HCC with M2 status was detected. The mass has a Max/Min-R of 1.59 and heterogeneous moderate hyperintensity on T2WI **(I)**, which displays no or minimal enhancement (red arrow) on AP, PVP, and DP(J-L). The lesion appears as extranodular nodule (black arrowhead) **(L)**.

**Figure 2.**
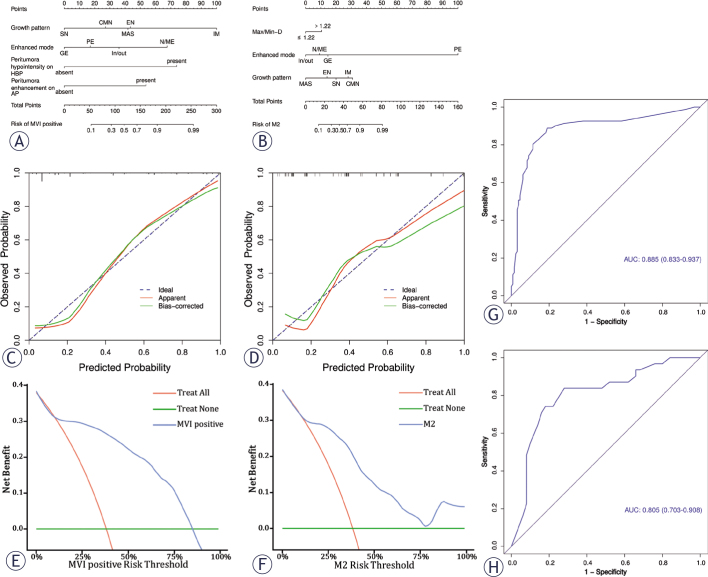
(**A,B**) The MVI nomogram was built by incorporating four variables, and among the MVI-positive cases, three variables were used to establish another nomogram for predicting M2 grade. **(C,D)** Calibration curves of the nomogram in predicting MVI and its M2 grade. X-axis, nomogram-predicted probability of MVI or M2; Y-axis, observed MVI or M2. **(E,F)** Nomogram model (blue line) outperforms all (red line) and none (horizontal green line) across all reasonable threshold probabilities in predicting MVI and its M2 grade. **(G,H)** The ROC curves demonstrate the discriminatory ability of the two nomograms of MVI positive and its M2 grade.

The presence or absence of these findings was recorded by two radiologists independently. The two readers maintained an interval of more than 2 weeks in images assessing for reducing recall bias. Discordant results were settled by consulting a senior radiologist and finally reached a consistent conclusion.

### Laboratory and histopathology data evaluation

The clinical and laboratory parameters assessed included patient information, liver disease etiology, and critical laboratory test results (alpha-fetoprotein [AFP], hepatitis B surface antigen [HBsAg], hepatitis B e antigen [HBeAg], status of HBV or HCV infection, protein induced by vitamin-K absence or antagonist II [PIVKA-II]). To standardize the distribution of some variables, we converted some values using lg10 when appropriate.

The histologic parameters of the resected specimen included: tumor size, the Edmondson-Steiner grade (classified into I to IV), satellite nodules, the status of MVI, tumor necrosis, liver cirrhosis, grade of hepatitis activity, stage of liver fibrosis (the METAVIR scoring system), capsule formation (with or without disruption). All histopathology samples were performed on the three-grade MVI grading (MVI-TTG) scheme from the less bleeding and necrotic sections. MVI was characterized as the presence of tumor cells that remain visible only under microscopy in portal veins, hepatic veins, and tumor capsule vessels through the endothelium.^4^ Samples were classified as M0 (no MVI), M1 (MVI sites no more than 5 and lie within 1cm of the periphery of the tumor), and M2 (MVI > 5 or farther than 1 cm from the tumor fringe)^4^, both M1 and M2 were classified as MVI-positive (M1/2).

### Statistical analysis

Continuous parameters were presented as means ± standard deviations or medians with interquartile ranges, and categorical values were expressed as numbers (percentages). Categorical data were compared using Fisher’s exact test or Chi-Square test. Partial continuous variables were altered to categorical or ranking variables based on clinical relevance ranges or tiered systems for greater clinical utility and model simplicity. Comparisons for continuous data were carried out according to distribution using ANOVA or Mann-Whitney U tests, as appropriate. Univariable and multivariable logistic regression analyses were conducted to identify factors related to MVI status. Multivariate logistic regression analysis results were implemented to build MVI and its M2 grade prediction nomograms and sensitivity, specificity, positive predictive value (PPV), negative predictive value (NPV), and accuracy were calculated. The area under the curve (AUC) and calibration curves were used to evaluate the nomograms’ prediction capability and accuracy, respectively. A two-tailed p-value lower than 0.05 was defined as statistically significant. All statistical analyses were performed using SPSS version 23.0 (SPSS Inc, Chicago, IL, USA) and R version 4.2.2. We converted AFP and PIVKA-II into lg10 alteration to make their distributions tend to be normal.

## Results

### Clinicoradiologic characteristics for assessing MVI grades

213 patients (57.4 ± 0.8 years, 177 male patients) with a median age of 58 years (range, 27–85 years), from two medical centers who satisfied the inclusion criteria and enrolled during the study period (Supplementary Figure 1). The detailed characteristics of patients are presented in Supplementary Table 2. The most predominant cause of the underlying liver disease was hepatitis B viral infection (157 of 213 patients, 73.7%). 135 patients (63.4%) were suffering from liver cirrhosis. According to the histology, the severity of MVI was ranked as M0, M1, and M2, in 132 (63.4%), 50 (23.5%), and 31 (14.6%) patients, respectively. The MVI-positive group (M1/2) exhibited statistically larger tumor sizes, considerably higher levels of AFP value, and more likely in the presence of satellite nodules, irregular shapes, vague margins, bird’s beak sign, peritumoral enhancement on AP, and peritumoral hypointensity on HBP (all p < 0.05) in contrast to the M0 group. Furthermore, compared to the M0 group, a greater proportion of patients with III-IV grades HCC was seen in the M1/2 group (P = 0.019) (Supplementary Figure 2). In M1 and M2 subtypes, the more severe the MVI grade, the higher the rate of larger the tumor size, the Max/Min-R in tumor diameter, and the presence of vague margins (Supplementary Table 2). Groups M0 and M1/2 as well as groups M1 and M2 showed statistically different enhancement modes (P = 0.03, and P = 0.008, respectively) and growth patterns (P < 0.001, and P = 0.04, respectively) (Supplementary Table 2). No other radiologic variables were found to have discernible differences in assessing MVI grades (Supplementary Table 2).

### Correlations between clinicoradiologic features and MVI grades

Table 1 and Table 2 displayed the results of univariate and multivariate analyses of risk factors connected to MVI status. Univariate analyses showed that twelve variables, specifically AFP ≥ 20ng/mL, satellite nodule, III-IV grades of Edmondson-Steiner, Max/Min-R, irregular tumor shape, nonsmooth tumor margin, non-solitary growth morphologies, no/mini enhanced mode, beak sign, peritumoral enhancement on AP, and peritumoral hypointensity on HBP were significantly correlated when comparing clinicoradiologic parameters between M0 and other degrees of MVI (both M1 and M2) (all p < 0.05). The univariate analysis’s results were striking in that when MVI existed they showed a substantial correlation between the M2 grade and four variables: Max/Min-R, non-smooth tumor margin, confluent multinodule growth type, and non-washin/washout enhanced modes. Furthermore, multivariate analysis revealed the following independent risk factors for the presence of MVI: non-solitary growth types, no/mini enhanced mode, peritumoral enhancement on AP, and peritumoral hypointensity on HBP; the independent variables for MVI in M2 grade were linked to the Max/Min-R, confluent multinodule growth type, and non-washin/washout enhanced modes (Table 1, Table 2).

**TABLE 1.** Univariate and multivariate logistic regression analysis for predicting MVI-positive HCCs

Variable	Univariable analysis		Multivariable analysis	
	Odds ratio (95 % CI)	P-value	Odds ratio (95 % CI)	P-value
**Clinical features**				
Age (≥ 57 years)	1.11 (0.64–1.94)	0.702		
Sex (male)	1.28 (0.60–2.72)	0.525		
Etiology (hepatitis B virus)	1.02 (0.54–1.93)	0.960		
AFP (≥ 20ng/mL)	1.98 (1.12–3.49)	0.018*		
PIVKA-II lg10	1.18 (0.84–1.66)	0.342		
**Pathological data**				
Edmondson-Steiner (III-IV)	2.35 (1.05–5.26)	0.037*		
Satellite nodule	2.53 (1.43–4.48)	0.001*		
**MRI findings**				
Tumor Max-D (≥ 3.9cm)	2.14 (1.22–3.77)	0.008*		
Tumor Min-D (≥ 3.1cm)	2.04 (1.16–3.59)	0.014*		
Irregular tumor shape	4.59 (2.54–8.33)	<0.001*		
Non-smooth tumor margin	2.93 (1.63–5.27)	<0.001*		
Solitary nodule growth	0.18 (0.10–0.34)	<0.001*	0.25 (0.12–0.52)	< 0.001*
No/mini enhanced mode	2.76 (1.31–5.85)	0.008*	3.24 (1.19–8.88)	0.022*
Beak sign	4.07 (2.25–7.38)	<0.001*		
Arterial Rim-enhancement	2.07 (0.97–4.42)	0.060		
Peritumoral enhancement on AP	5.23 (2.56–10.66)	<0.001*	5.19 (2.15–12.53)	< 0.001*
Peritumoral hypointensity on HBP	11.26 (5.84–21.68)	<0.001*	10.74 (5.07–22.75)	< 0.001*

1 AFP = alpha-fetoprotein; AP = arterial phase; CI = confidence interval; HBP = hepatobiliary phase; MRI = magnetic resonance imaging; MVI = microvascular invasion; OR = odd ratio; PIVKA-II = protein induced by vitamin K absence or antagonist-II; Tumour Max-D = maximum diameter of tumor; Tumour Min-D = minimum diameter of tumor. The asterisked entries indicate P < 0.05.

**TABLE 2.** Univariate and multivariate logistic regression analysis for predicting M2 grade

Variable	Univariable analysis		Multivariable analysis	
	Odds ratio (95 % CI)	P-value	Odds ratio (95 % CI)	P-value
**Clinical features**				
Age (≥ 58 years)	1.68 (0.68–4.14)	0.262		
Sex (male)	1.27 (0.35–4.69)	0.703		
HBeAg(+)	0.32 (0.09–1.05)	0.060		
AFP lg10	1.86 (0.75–4.63)	0.183		
PIVKA-II lg10	1.09 (0.64–1.88)	0.745		
**Pathological data**				
Satellite nodule	0.46 (0.18–1.16)	0.098		
**MRI findings**				
Tumor Max-D (≥ 4.3cm)	1.39 (0.56–3.42)	0.480		
Tumor Min-D (≥ 3.7cm)	1.07 (0.44–2.61)	0.888		
Max/Min-R (≥ 1.22)	3.53 (1.38–9.04)	0.009*	2.91 (1.07–7.92)	0.036*
Irregular tumor shape	1.32 (0.52–3.32)	0.560		
Non-smooth tumor margin	3.43 (1.33–8.82)	0.011*		
Confluent multinodule growth	3.29 (1.19–9.08)	0.021*	3.92 (1.25–12.25)	0.019*
Washin/washout enhanced mode	0.34 (0.13–0.88)	0.026*	0.31 (0.11–0.92)	0.035*
Beak sign	1.18 (0.48–2.91)	0.721		
Arterial Rim-enhancement	2.15 (0.73–6.34)	0.167		
Peritumoral enhancement on AP	1.99 (0.79–5.01)	0.143		
Peritumoral hypointensity on HBP	1.77 (0.63–4.92)	0.227		

1 AFP = alpha-fetoprotein; AP = arterial phase; CI = confidence interval; HBP = hepatobiliary phase; MRI: magnetic resonance imaging; MVI = microvascular invasion; Max/Min-R = Tumour Max-D/Tumour Min-D; OR = odd ratio; PIVKA-II = protein induced by vitamin K absence or antagonist-II; Tumour Max-D = maximum diameter of tumor; Tumour Min-D = minimum diameter of tumor. The asterisked entries indicate P < 0.05.

### Nomogram model establishment and validation

The nomograms were established based on the aforementioned independent risk factors, and one combined model integrated non-solitary growth morphologies and no/mini enhanced mode with peritumoral enhancement on AP and peritumoral hypointensity on HBP in predicting MVI-positive with an AUC of 0.885 (95% CI: 0.833–0.937), and another combined model including the Max/Min-R exceeding 1.22, confluent multinodule growth type and non-washin/washout enhanced modes could predict M2 grade with an AUC of 0.805 (95% confidence intervals [CI]: 0.703–0.908). Figure 2A and 2B show the nomograms for the predictions of MVI-positive and M2 grade. The probability of MVI and M2 grade in HCC can be easily estimated by summing the points of the variables and locating the corresponding score on the probability axis. We employed calibration curves to assess the consistency between the expected probability and the actual observed values of the models for MVI and M2 grade, taking into account the importance of MVI status for clinical diagnosis and therapy (Figure 2 C,D). The calibration curves (Figure 2 C,D) show reasonably good consistency between the estimated probability of the nomograms and the MVI-positive and its M2 grade. This implies that our nomogram-based therapy approach will lead to better clinical outcomes. Referring to Figure 2 E,F, the DCA demonstrated that the nomogram prediction models produced a greater net clinical benefit in predicting MVI and its M2 grade than the “treatall” or “treat-none” approaches. Detailed data on the performance of combined models for diagnosing MVI grade were recorded in Table 3, and the AUC analyses were presented in Figure 2 G,H.

**TABLE 3:** The diagnostic performance of nomogram models for MVI-positive and M2 grade in HCC

Model	AUC (95%CI)	Accuracy (%)	Sensitivity (%)	Specificity (%)	PPV	NPV
**MVI-positive**	0.855(0.833–0.937)	84.0	88.9	81.1	74.2	92.2
**M2 grade**	0.805(0.703–0.908)	79.0	74.2	82.0	71.9	83.7

1 Data are percentages, data with 95% confidence interval (CI) in parentheses; AUC = area under the receiver operating characteristic curve; HCC = hepatocellular carcinoma; MVI = microvascular invasion; NPV = negative predictive value; PPV = positive predictive value

## Discussion

MVI occurrence indicates aggressive tumor biology and is attributed to an unfavorable prognosis. Even with a methodical therapy, evaluation of MVI grading continues to be a challenge and affects personalized care.^23^ However, most researchers simply utilized a binary classification method to predict the presence of MVI, without considering MVI grading, particularly M2 grade. In terms of the prognosis for HCC, M2 status is more likely to experience recurrence, intrahepatic metastases, and residual tumor than M1 status.^24^ Therefore, we extract enhanced modes and growth patterns of tumors from MRI to provide a universal, noninvasive, and robust method for preoperative patient prediction of MVI status. In our study, accuracy for M2 grade diagnosis is up to 79%, with corresponding sensitivity and specificity of 74% and 82%, respectively.

Considering earlier publications^25^, serum AFP level was significant risk factor for MVI existence and are associated with factors of tumor aggressiveness, including early recurrence.^26^ In this study, the serum AFP level was significantly related to MVI occurrence, even when the AFP value was slightly above the limit, but without significant statistics in predicting M2 grade. The fact that AFP is not a particular marker for HCC and can remain unstable under a range of circumstances could be the cause. For Chen’s results, HCCs with M1 or M2 grade may have different high AFP level and lack significance^6^, which matches our findings. Based on the Milan criteria, a tumor greater than 5 cm is ineligible for liver transplantation, however, MVI-positive HCCs less than 5 cm may lose the second chance in transplant and get worse outcomes. Nonetheless, varying research produced tumor size in diverse critical values when assessing the prediction of MVI. Most researchers proposed a tumor size greater than 5.0 cm as a general visual predictor and MVI status positively correlates with tumor size.^8^ According to Shirabe *et al*.^27^ a tumor size exceeding 3.6 cm was considered to be a reliable indicator of MVI. In our search, a tumor size larger than 3.9 cm was regarded to be a sign of MVI. In this study, it is worth considering that the P value of HBeAg was close to 0.05 in univariate analysis, and in Jian Liu’s study^28^, patients with HBeAg positive were an independent factor of MVI-positive and associated with a poorer prognosis. Given the similarity of our results, we suppose that positive HBeAg will probably be considered as a potential factor for assessing the M2 grade with further sample enlargement.

The previous study indicates lesions expressed in morphological changes are more easily discovered and acceptable than other complex enhancement and signal features in clinical diagnosis.^26^ Adjacent non-tumor tissue was thought to be the region where HCC cells frequently spread into the surrounding liver tissues, causing uneven tumor surface and irregular growth^8^, implying the occurrence of MVI, which was consistent with our findings. Li Y *et al*.^29^ applied the tumor surface inclination angles to assess HCCs with MVI presence, which had a higher surface irregularity rate and worse sphericity. In this study, we utilized the Max/Min-R as a substitute for flatness, as done in Zheng’s^8^ study, to serve as an easily implemented assessment method for tumor irregularity instead of preoperative 3D morphological evaluation. The latter cannot be routinely performed in the clinic. Our research further refined and categorized the marginal morphological features to evaluate the distinctive growth pattern of M2.^20,29^ Our findings indicate that a Max/Min-R exceeding 1.22 suggests a more severe irregularity and is substantially correlated with a high-risk factor for M2, which aligns with the results reported in Zheng’s study. Among the MVI-positive nomogram predictors, the immersed growth had the highest score (100 points on the scale axis). For the M2 status nomogram, confluent multinodule and immersed growth had a higher score for M2 status. Wang X *et al*.^30^ illustrate that HCC with irregular shape or rough edge indicating multiple microscopic layers budding at the periphery of the lesion is more prone to induce tumors to grow in an immersed or multinodular manner.

Among the image features that were repeatedly verified and recognized, some specific features of the peritumoral region corroborate the MVI presence, including arterial rim-enhancement, arterial peritumoral enhancement on AP, and peritumoral hypointensity on HBP. The aforementioned features of the tumor-liver region may suggest abnormal hemodynamics in adjacent tissues caused by extracellular matrix disarray, tissue distortion, tumor cell blockage, cell proliferation, and inflammation.^8,31^ These criteria are significant in differentiating MVI-positive in clinical importance and align with our findings. Previous research has categorized the enhancement modalities for HCC into typical and atypical patterns in assessing MVI occurrence.^32–35^ However, they may not fully distinguish between M1 and M2 as identified in the research.

Intratumor vascularity is frequently utilized as an imaging marker for aggressive HCC and displays various enhanced patterns, and some studies have been reported to be associated with MVI occurrence, early relapse, and recurrencefree survival.^33,36–38^ Hence, we can assess indirectly inner-tumor vessel clusters by observing the enhancement pattern. In this study, we divided the enhancement patterns into four categories, and MVI-positive HCCs are more likely to exhibit the no/minimal enhancement pattern, which could indicate hypoperfusion as a result of tumor cell blockage in the peritumoral area of the microvas-culature.^39^ Likewise, the promising results from Li Zhang *et al*.^36^ analyzed the triphasic CT enhancement images by hemodynamics software and showed that the presence of MVI had lower hemodynamic perfusion parameters, which parallels what we observed. While HCCs with M2 grade exhibit a higher propensity for developing persistent enhanced mode and are unlikely to have washin/washout enhanced mode. This may be attributed to the existence of vessels encapsulating tumor clusters, or indirectly resulting in persistent enhancement.^33^ Or maybe peri-tumoral microvascular branches were disrupted or obstructed by the tumor cells and caused compensatory hyperperfusion in subsequent phases.^8^

By adding tumor morphology and enhancement modes to the nomogram, we improved our study’s clinical applicability while raising the MVI grading accuracy. Yumeng Li *et al* developed an MVI prediction model based on integrating laboratory indicators and 3D morphological features, yielding an AUC of 0.831^29^, which could potentially improve the predictive efficiency. Although our study used two-dimensional morphological features to assess tumor growth patterns, the nomogram showed satisfactory performance in identifying the presence of MVI and its M2 status. Based on the calibration curves, both nomograms showed good consistency between the observed probability and the predicted probability. Further evidence of the two nomograms with great clinical value came from DCA, which demonstrated a net benefit of the nomograms with a risk threshold greater than 0.15. In addition, radiologists or surgeons can quickly sense the features and scores from these in clinical practice, even if the variations in scanning parameters and apparatus.

Our study skipped over the clinical imaging features used for M1 status or developing a model to differentiate it. Due to the existence of similar issues in other models^8,40^, standardizing the clinical model of M1 was not so functional. We also omitted pathology features from the multivariate analysis since they depend on an invasive exam. Adding histopathologic features would have compromised the model’s validity and financial viability. Despite their potential, radiomics and deep learning both have high labor requirements and poor interpretability; they also require numerous samples, higher-quality imaging, and scanning conditions.^41^ Most institutes lack data processors and technicians to process. Compared to our nomogram model, it is not as practical and convenient for clinical use.

Several limitations of our study should be noted. Initially, there can be selection bias due to the retrospective design of this research. Additionally, the patient sample size was sufficient, but somewhat in certain groups, the amount was limited due to the multi-characteristics and the dispersed distribution of the cases, although we emerged the patients from two centers and discarded the group validation. Furthermore, our study solely focused on analyzing the appearance of tumor morphology and enhancement; in contrast, the utilization and analysis merger of laboratory tests (AFP, PIVKA-II) in predicting MVI grades were weakly relevant. Lastly, there is no concrete evidence establishing a direct correlation between radiological features and MVI grades. Future large dataset integration, multi-parameter combining, prospective multicentre trials, and independent validation would be required to investigate the relationship between radiological features and MVI grade.

In conclusion, we incorporated easy-to-recognize features and established nomograms to enable satisfying MVI status prediction. When clinicians consider factors and score the nomogram systems to deem the patient at high risk of MVI or maybe categorized as M2 grade, monitoring plan selection and personalized therapy design are helpful for HCC patients to start treatment in an efficient and curative way.

## Supplementary Material

Supplementary Material Details
