# Supplementary material for: Evaluating the severity of microvascular invasion in hepatocellular carcinoma, by probing the combination of enhancement modes and growth patterns through magnetic resonance imaging
Source: Radiol Oncol. 2025 Apr 11;59(2):183–92. doi: 10.2478/raon-2025-0021 (PMC12182925; doi:10.2478/raon-2025-0021)
Supplement: Supplementary file 1 — Supplementary Material Details [file raon-2025-0021_sm.pdf]

Evaluating the severity of microvascular invasion in hepatocellular carcinoma, by probing the combination of enhancement modes and growth patterns through magnetic resonance imaging

Yanzhuo Li, Sijie Li, Yan Lei, Lianlian Liu, Bin Song

doi: 10.2478/raon-2025-0021

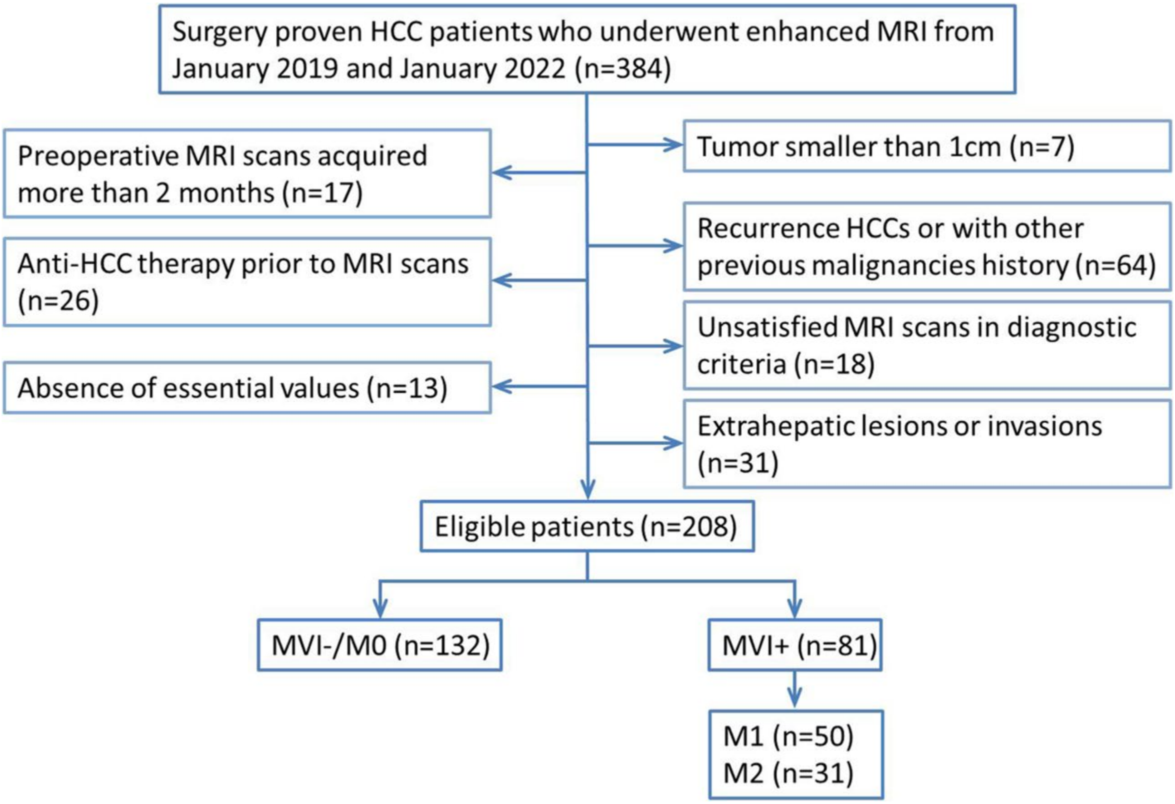

SUPPLEMENTARY FIGURE 1. Diagram displays the inclusion and exclusion criteria for this study.

HCC = hepatocellular carcinoma; MRI = magnetic resonance Imaging; MVI = microvascular invasion

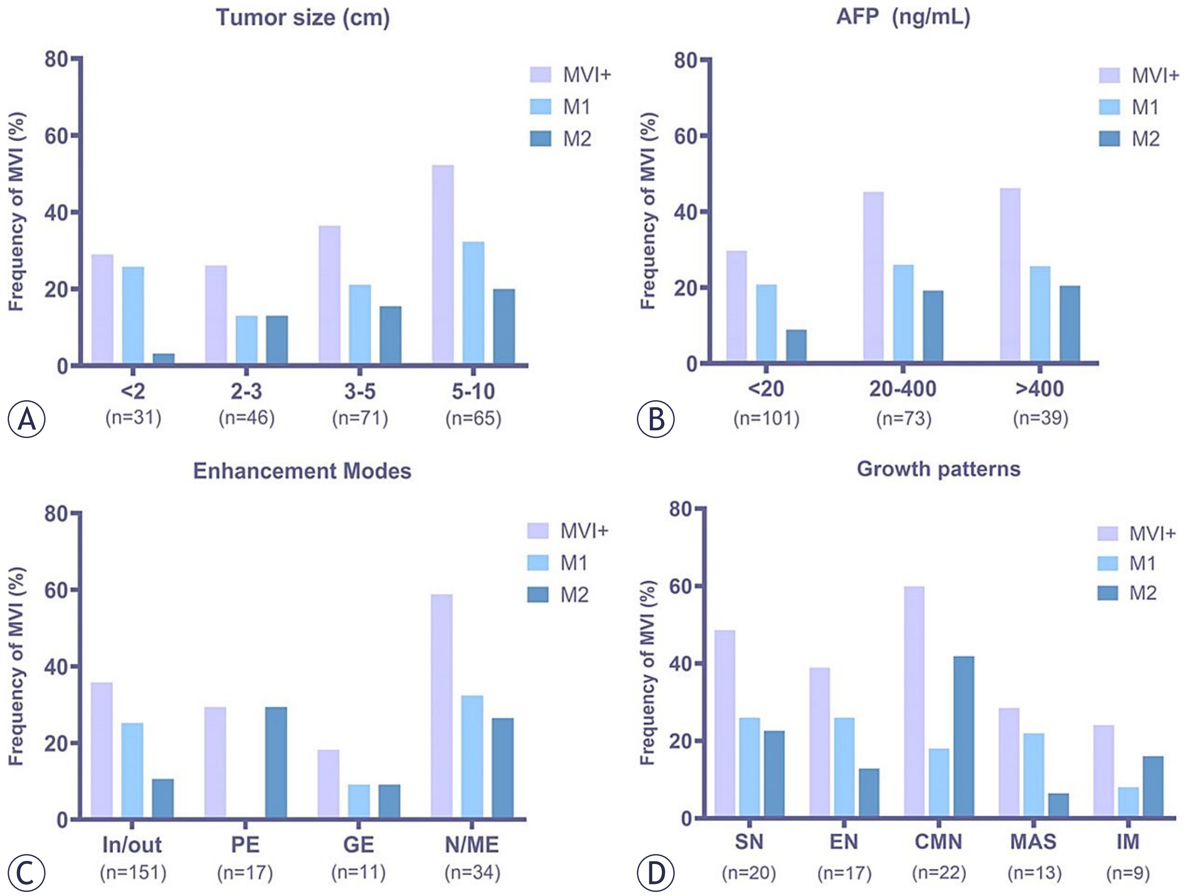

SUPPLEMENTARY FIGURE 2. Frequency of microvascular invasion status based on tumor size **(A)**, AFP level **(B)**, Enhancement modes **(C)**, and Growth patterns **(D)**.

AFP = alpha-fetoprotein; CMN = confluent multinodule; EN = extranodular nodule; GE = gradual enhanced; In/out = washin and washout; IM = immersed;MAS = massive; MVI = microvascular invasion; N/ME = no/minimal enhanced; PE = persistent enhanced; SN = solitary nodule
